# Bacterial peritonitis in paediatric appendicitis; microbial epidemiology and antimicrobial management

**DOI:** 10.1186/s12941-023-00591-1

**Published:** 2023-06-03

**Authors:** Keir Bhaskar, Simon Clarke, Luke S. P. Moore, Stephen Hughes

**Affiliations:** 1 Chelsea and Westminster NHS Foundation Trust, 369 Fulham Road, London, SW10 9NH UK; 2 North West London Pathology, Imperial College Healthcare NHS Trust, Fulham Palace Road, London, W6 8RF UK; 3 National Institute for Health Research Health Protection Research Unit in Healthcare Associated Infections and Antimicrobial Resistance, Imperial College London, Hammersmith Campus, Du Cane Road, London, W12 0NN UK; 4 Department of Medicine, Imperial College London, Exhibition Road, South Kensington, London, SW7 2BX England, UK

**Keywords:** Microbiology, Paediatrics, Surgery, Apendicitis

## Abstract

**Background:**

Appendicitis remains a common surgical emergency in children. Empirical antibacterial treatment is indicated to reduce infective complications. We investigate the bacterial pathogens identified intra-operatively during appendectomies in children to guide empirical surgical antimicrobial prophylaxis options.

**Methods:**

A retrospective analysis of patients (< 18 years old) undergoing an appendectomy across a multisite London hospital (Nov 2019–March 2022) was undertaken. Patient-related outcomes including length of hospital stay (LOS), days of antibacterial therapy (DOT), intra-operative microbiology and post-operative radiology reports were interrogated.

**Results:**

304 patients underwent an appendectomy during this period; 39.1% of patients had intraoperative samples cultured. Bacterial pathogens were found in 73/119 (61.3%) cases; the most common isolates being *Escherichia coli* (42.0%), *Pseudomonas aeruginosa* (21.0%), milleri *Streptococcus spp. *(14.3%) and *Bacteroides fragilis* (5.9%). Polymicrobial infection was common (32/73). Isolation of *Pseudomonas *spp*.* from intra-operative sampling was associated with a greater LOS (7.0 vs. 5.0 days; p = 0.011) but nil effect on the incidence of postoperative collections. Presence of milleri *Streptococcus spp.* was associated with longer LOS (7.0 vs. 5.0 day; p = 0.007), DOT (12.0 vs. 8.5 day; p = 0.007) but had no observed outcome on postoperative collections (29.4% vs. 18.6%; p = 0.330). 48% of *E. coli* positive cultures were co-amoxiclav resistant and prolonged LOS compared to the non-resistant group (7.0 vs. 5.0 days; p = 0.040) but had no difference in post-operative collections (29.2% vs. 17.9%; p = 0.260).

**Conclusion:**

A high proportion of children with appendicitis have *Pseudomonas *spp*.* isolated, leading to a prolonged LOS. Evolving Enterobacterales resistance and the presence of *Pseudomonas *spp*.* necessitate extended antibacterial coverage for paediatric appendectomies with evidence of peritonitis.

## Introduction

Acute appendicitis is the most common surgical emergency and cause of abdominal pain in children [1–3]. The estimated lifetime risk is around 12% and 25% for males and females, respectively [1]. Appendicitis can occur at any age; with the peak incidence ocurring between the ages of 10–19 years old [1, 2]. While younger children experience longer hospital stays, higher rates of readmission and are more likely to present with perforation, older children tend to develop intra-abdominal collections [4, 5]. Antibacterial therapy usually in combination with surgical intervention is key to optimising outcomes.

Bacterial overgrowth is present in acute appendicitis; the most common bacterial isolates consist of *Escherichia coli, Bacteroides fragilis, milleri Streptococcus spp., Peptostreptococcus *spp*., and Pseudomonas *spp. in their order of prevalence [6, 7]. However, their role in pathogenesis of appendicitis has been debated. The anaerobe *B. fragilis* is found in increased concentrations in acutely inflamed appendices, suggesting some causal connection with the inflamed intestinal mucosa but no definitive pathogenesis has been confirmed [8, 9]. Milleri *Streptococcus spp.* have a similar causal relationship in appendicitis but it has been confirmed to be associated with intra-abdominal abscess formation [10–12]. Enterobacterales that colonise the gastro-intestinal tract, such as *E. coli*, likely cause opportunistic infection secondary to local inflammation and/or perforation with appendicitis [13]. *Pseudomonas aeruginosa* has been described as the critical pathogen isolated in perforated and gangrenous paediatric appendicitis, although its role in adults is rare [9, 14]. The accumulating evidence determining the roles of these bacteria in complicating postoperative cases highlights the need for an empirical antibacterial regimen with activity against these common pathogens.

National guidelines within the UK for the surgical management of paediatric appendicitis favour beta-lactam based antibacterial prophylaxis; co-amoxiclav or cephalosporin based therapies are commonly recommended with antipseudomonal agents not routinely included [15]. Limited study data is available to support these treatment recommendations and as Enterobacterales resistance increases and postoperative infections complicated by *P. aeruginosa* become more frequent,many centres may extend empirical coverage beyond the national recommendations [16]. In our local organisation, we combine an aminoglycoside (gentamicin) with co-amoxiclav (or cefuroxime plus metronidazole in penicillin intolerant patients) to extend antibacterial coverage for common Enterobacterales resistance mechanisms and for antipseudomonal activity. Alternative options may include using antipseudomonal beta-lactams [17, 18].

Optimising peri-appendectomy antibacterial prescribing can improve patient outcomes while balancing the antimicrobial stewardship priorities. Identifying patient groups at risk of developing postoperative collections, the impact of milleri *Streptococcus spp.* and *P. aeruginosa* on infective complications and the optimum empirical antibacterial prophylaxis activity and duration remains unknown for our paediatric patients undergoing appendectomy [11, 12, 19]. This retrospective study aims to determine the microbial epidemiology of paediatric appendicitis from intra-operative cultures in order to assess the appropriateness of empirical antimicrobial prophylaxis during surgery.

## Methods

### Study setting and design

A retrospective observational analysis was undertaken of paediatrics patients between the ages of 1 and 18 years of age who underwent appendectomy in a large single centre NHS acute Trust; Chelsea & Westminster NHS hospital (London, UK). The study included all presentations with diagnostic coded acute appendicitis cases or surgical appendectomy between November 1st 2019 and March 1st 2022. Data was obtained from the electronic health record platform (Cerner^®^Missouri, United States) including patient characteristics, type of appendicitis (simple or complex), type of surgery (laparoscopic or open), postoperative imaging and interventions, intraoperative cultures (if any), length of hospital stay in days (LOS), 30-day readmission, radiologically defined postoperative collections, days of antibacterial therapy [DOT], and antibacterial treatments received. Children who underwent an interval appendectomy, had primary surgery at another centre, had non-surgically managed appendicitis or had incomplete patient records detailing their surgery were excluded. Intra-operative cultures were not mandatory but were morecommon in children with intra-operative evidence of peritonitis.

### Laboratory techniques

Microscopy for causative pathogens was investigated in line with the national UK Standards for Microbiology Investigations from Public Health England on the relevant media, atmospheres and duration noted in the relevant standard operating procedure [20]. Isolate speciation was performed using MALDI-TOF spectroscopy (Biotyper^®^, Bruker). Antimicrobial susceptibilities were determined by disc diffusion using European Committee on antimicrobial susceptibility Testing (EUCAST v.10) criteria [21].

### Statistical analysis

Statistical analysis was carried out using GraphPad Prism 9. Normality was assessed using a Kolmogorov-Smirnov test. Parametric data was presented using the mean and its respective standard deviations and, a student t-test was used to determine statistical significance. Non-parametric descriptive data was presented as median values with interquartile ranges (IQR). The Chi-Squared and Fisher’s exact test was used to compare categorical data and a Mann-Whitney U test was performed for continuous data. Post-appendectomy outcomes for each bacteria isolate were compared to culture negative outcomes, anda value of p < 0.05 was considered statistically significant.

#### Study approval

The project was defined as a service evaluation by the Chelsea and Westminster NHS Foundation Trust R&D department and assigned approval (reference CSS093).The information was collected as part of routine work by the infection team and the need for individual consent was waived for this retrospective analysis following review by the Chelsea and Westminster NHS Foundation Trust Research & Governance Office. All data was collected and stored in concordance with the Data Protection Act and the General Data Protection Regulation.

## Results

**Fig. 1:**
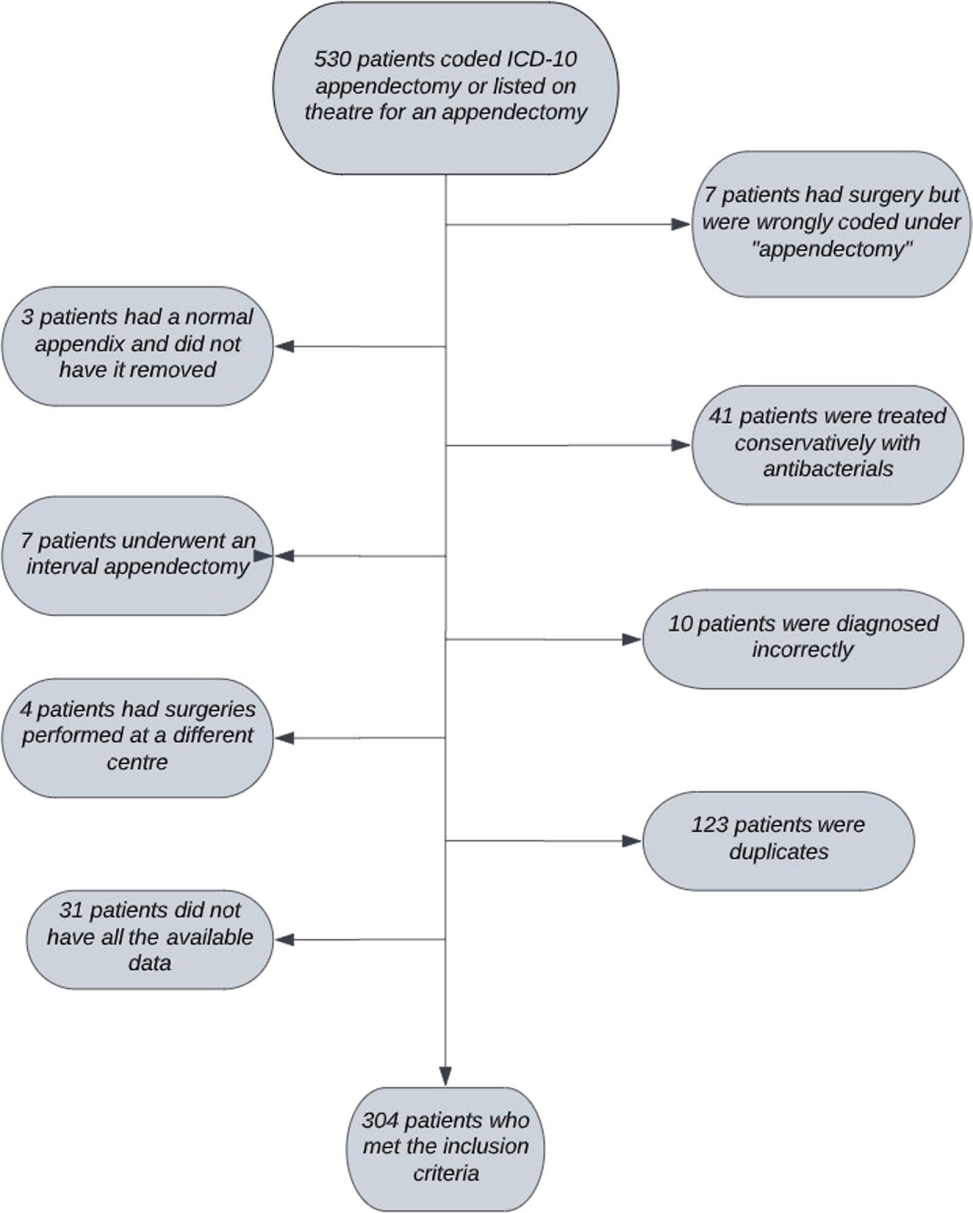
A flowchart demonstrating the number of patients that were excluded from the raw data cohort to achieve the final cohort who met the inclusion criteria

A total of 530 patients admitted to the Chelsea & Westminster NHS Trust (November 2019–March 2022) with either appendectomy coded via ICD-10 or listed on theatre as having had an appendectomy performed. 304/530 patients were included in the final analysis (Fig. 1); the median age was 11 years old (IQR 8–14) and 177/304(58.2%) were male. Laparoscopic appendectomy 283/304(93.1%) was the most frequent intervention, with 18/304(5.9%) and 3/304(1%) having an open surgery and laparoscopic procedure converted to open, respectively. The median LOS was 3 days (IQR 2–6) and DOT was 6 days (IQR 2–9). The overall rate of readmission and postoperative collections was 29/304(9.5%) and 32/304(10.5%), respectively.

### Microbiological findings

Intra-operative cultures were taken for 119/304 (39.1%) patients and 73/119 (61.3%) of patients had one or more pathogens cultured; 32/73 (43.8%) cultures were polymicrobial. The most common organism isolated from cultures during an appendectomy was *E. coli* 50/119 (42.0%). The presence of pathogens was more commonly identified in patients who developed postoperative collections than those without (87.5% vs. 54.3%, p < 0.01).

Children with intra-operative cultures performed had a longer LOS (6 days vs. 2 days, p < 0.0001) and longer DOT (9 days vs. 3 days, p < 0.0001) (Table 1). This group had higher rates of readmission (14.3% vs. 6.7%, p = 0.045) and radiologically confirmed postoperative collections (20.2% vs. 4.5%, p < 0.0001) compared to the non-cultured group. Median age of the culture group was found to be lower 10 years (7–13) vs. 12 (8.5–15), p < 0.0001.

**Table 1:** A comparison between the group with intraoperative cultures and the group without cultures and their effects on length of stay, days of antibacterial therapy, 30-day readmission, postoperative collections and postoperative interventions

	Intraoperative culture sent (n = 119)	No culture sent (n = 185)	Statistical comparison
Median age, years (IQR)	10 (7–13)	12 (8.5–15)	p < 0.0001
Male sex, n (%)	69 (58.0%)	108 (58.3%)	
Median LOS, days (IQR)	6 (3–9)	2 (2–3)	p < 0.0001
Median DOT, days (IQR)	9 (7–11)	3 (1–7)	p < 0.0001
30-day readmission, n (%)	17 (14.3%)	12 (6.7%)	p = 0.045
Postoperative collections, n (%)	24 (20.2%)	6 (4.5%)	p < 0.0001
Intervention of collection, n			
Conservative (Continued antibacterials post-operatively)	15	4	
Drain	7	1	
Aspiration	2	1	
Microbiology results, n			
*E. coli* isolated	50		
Milleri *Streptococcus spp.* isolated	17		
*Pseudomonas aeruginosa* isolated	25		
Anaerobes isolated	17		
Polymicrobial infection present	32		

Polymicrobial infection was defined as 2 or more bacterial pathogens isolated from intra-operative samples

i. *E. coli*

*Escherichia coli* was the most frequently isolated pathogen (Table 2). Patients with intra-operative cultured *E. coli* endured a greater LOS (8 days vs. 5 days, p < 0.0001) and duration of antibacterial treatment (DOT 11 days vs. 7 days, p < 0.0001) than the control group of non-*E. coli* cultured intra-operative cases. No difference in rates of readmission (8/50; 16.0% vs. 9/69; 13.0%, p = 0.79) or presence of postoperative collections (12/50; 24.0% vs. 12/69; 17.4%, p = 0.49) were observed.

**Table 2:** A list of the frequency of the most common cultured organism combinations and the percentage collections formed from this sample type

Organisms cultured	Number of samples (n)	Number of collections formed (%)
*E. coli only *	24	7 (29.2%)
*E. coli & Pseudomonas aeruginosa*	10	1 (10%)
*Anaerobes* *only*	6	2 (33.3%)
*Pseudomonas only*	5	1 (20%)
*E. coli & anaerobes*	4	2 (50%)
*E. coli, milleri Streptococcus spp. & anaerobes*	4	2 (50%)
*E. coli & milleri Streptococcus spp.*	4	0 (0%)
*Pseudomonas aeruginosa & anaerobes*	3	1 (33.3%)
*Milleri Streptococcus spp. only*	3	1 (33.3%)
*E. coli & milleri Streptococcus spp., Pseudomonas aeruginosa*	3	0 (0%)
*Milleri Streptococcus spp. & Pseudomonas aeruginosa*	2	2 (100%)

In vitro susceptibility for cultured *E. coli* isolates in this study was 26/50 (52%), 45/50 (90%), 46/90 (92%) and 43/50 (86%) for co-amoxiclav, 3rd generation cephalosporins, ciprofloxacin and gentamicin, respectively. Resistance to first line empiric surgical prophylaxis (combination co-amoxiclav and gentamicin) was 8/50 (12%). The presence of co-amoxiclav resistant *E. coli* was associated with a longer LOS (7 days vs. 5 days, p = 0.04) than a control of co-amoxiclav susceptible *E. coli*.

ii. *Pseudomonas aeruginosa*

Patients with intra-operative cultured *Pseudomonas aeruginosa* had a greater LOS (7 days vs. 5 days, p = 0.011) compared to a control group of non*-Pseudomonas* cultured intra-operative cases. No differences in rates of re-admission (4/25; 16.0% vs. 13/94; 13.8%, p = 0.75) or postoperative collections (5/25; 20.0% vs. 19/94; 20.2%, p = 1.0) between the two groups were identified.

iii. *Milleri Streptococcus spp.*

Patients with intra-operative cultured  milleri *Streptococcus spp.* had a longer LOS (7 days vs. 5 days, p = 0.0069) and DOT (12.0 days vs. 8.5 days, p = 0.0070) compared to their non-*milleri Streptococcus spp.* controls. The rates of readmission (1/17; 5.9% vs. 16/102; 15.7%, p = 0.46) and rates of postoperative collections (5/17; 29.4% vs. 19/102; 18.6%, p = 0.33) did not differ significantly between the two groups.

iv. *Bacteroides fragilis*

Patients with intra-operative cultured *Bacteroides fragilis* had an increased risk of developing a postoperative collection (4/7; 57.1% vs. 20/112 17.9%, p = 0.03) and a trend to higher hospital readmissions (3/7; 42.9% vs. 14/112; 11.6%, p = 0.05) compared to patients with non-*Bacteroides fragilis* isolated.

### Antimicrobial prescribing

In this study, the most common empirical antibacterial regimen for intra-operatively culturd patients was co-amoxiclav and gentamicin, given to 68/119 (57.1%). Monotherapy with co-amoxiclav was the next common option, with 39/119 (32.8%) patients receiving this treatment. Other empirical regimens included a cephalosporin in combination with metronidazole or ciprofloxacin monotherapy.

Escalation of treatment usually consisted of piperacillin/tazobactam or meropenem with some patients requiring single doses of amikacin. Among the patients who tested positive for *E. coli*, 78% (39/50) received a combination of co-amoxiclav and gentamicin preoperatively. Of these patients, 26/50 (52.0%) had their initial antibacterial regimen altered, compared to 12/69 (17.7%) with non-*E. coli* pathogens cultured; this difference was statistically significant (p < 0.0001). Antibacterials were escalated in 15 out of 26 patients (57.7%) with *E. coli* infections, and 9 of these cases had co-amoxiclav resistance. A post-operative collection was identified in 12 children with *E. coli* positive cultures, all of these patients received a combination of co-amoxiclav and gentamicin preoperatively. Seven of these cultures where in vitro co-amoxiclav resistant but susceptible to gentamicin, and only one of these seven patients (who was gentamicin susceptible) required an escalation in antibacterial regimen.

For the *E. coli* positive patients that did not receive gentamicin preoperatively, none of them developed postoperative collections. However, in 10/11 (90.9%) of these patients, bacteria isolates identified were sensitive to their empirical treatment regimen.

Patients who tested positive for *Pseudomonas *spp*.* were more likely to have their initial antibacterial regimen changed (16/25; 64.0%) compared to patients with other pathogens (25/94; 26.9%, p = 0.0008). Intensification of regimen occurred in 10 out of 16 patients (62.5%) to achieve clinical stability, with only one case not receiving gentamicin pre-operatively. Escalation of treatment was uncommon within the *Streptococcus milleri* group [3 out of 17 patients (17.6%)].

## Discussion

This retrospective study evaluates the microbiology findings from intra-operative cultures in children undergoing appendectomy. The most commonly isolated pathogen is *E. coli*, and susceptibility to first-line beta-lactam co-amoxiclav is 52% (26/50), raising concerns about the reliability of this agent as monotherapy. Intra-operative culturing of *Pseudomonas *spp. is also frequently identified; however its pathogenicity remains unclear.

The role of intra-operative cultures in appendicectomies is widely debated [12]. At this local centre, it is commonly utilised for children with appendicectomies and evidence of peritonitis and /or systemic signs of sepsis. Some studies, such as the one by Foo et al., question the value of this practice, describing a low yield of pathogens (32%) and minimalistic changes to patient management [22]. Others argue that the increasing antimicrobial resistance and thus less reliable empiric treatment options necessitate the need for intra-operative cultures to identify these pathogens present and their resistance mechanisms [23, 24].

The presence of a cultured pathogen may not accurately reflect the problem pathogen at time of appendectomy which limits this and other similar studies. Selective culturing of our more complex cases (e.g. where pus is identified intra-operatively) may skew results for the more extreme presentations and not represent the microbiology of all patients [19]. From our findings, the presence of a confirmed pathogen is associated with post-operative complications including LOS and antimicrobial duration of therapy. However, many confounders exist. Once cultured, the presence of a pathogen can direct post-operative antibacterials through targeting resistance mechanisms to first-line therapies (e.g. ESBL Enterobacterales) or through extending beta-lactam therapy to cover *Pseudomonas.* Identification of milleri *Streptococcus spp.* resulted in prolonged DOT due to this pathogen’s association with abscess formation. Bespoke microbiology recommendations for extended treatment are common for milleri *Streptococcus spp.* associated complex intra-abdominal infections.

The availability of intra-operative samples is useful for guiding post-operative antibacterials, especially when complications such as post-operative collections exist. In our practice, consolidating empirical antimicrobials to a targeted therapy is common in our post-operative complicated cases to facilitate oral step down, prolonged intravenous antibacterials in the out-patient setting or escalation in clinically unstable cases.

This study confirms that *E. coli* is the most commonly isolated pathogen from intra-operative cultures (42.0%), similar to other epidemiological studies (32–85%) [18, 25]. As a common commensal organism within the intestinal lumen and known invasive pathogen, it is plausible that *E. coli* is responsible for postoperative infective complications and warrants empiric antibacterial coverage [13]. However, the increasing antimicrobial resistance of Enterobacterales, such as *E. coli*, is of increasing concern, and empiric antimicrobial treatments are becoming less robust. In this study, 48% of the *E. coli* positive specimens had phenotypic evidence of co-amoxiclav resistance. The addition of gentamicin extends activity against many of these resistant pathogens, with bespoke targeted therapy introduced post-operatively if complex infection persisted. This could be through use of ceftriaxone based options for non-ESBL infection or either ciprofloxacin or meropenem dependent on susceptibility data. Where co-amoxiclav resistant *E. coli* were present, the median LOS and DOT was approximately 48 h longer than a co-amoxiclav susceptible *E. coli* control. This may reflect delay to infection resolution or may be driven by prescriber behaviours for extended targeted therapy.

In children with postoperative infections requiring prolonged antibacterials, the presence of intra-operative culture resulted in a change of empiric therapy in 75% of patients. Addition of anti-pseudomonal beta-lactam where *Pseudomonas aeruginosa* was isolated, changing from co-amoxiclav where resistance confirmed or extended therapy for milleri *Streptococcus spp. *was common. Milleri *Streptococcus spp.* are a common coloniser of the gastrointestinal tract recognised for its virulent and invasive role, especially in acute appendicitis [10]. Various studies performing microbiological cultures have described a wide range of rates from 13 to 61%, with our study reporting 14.3% of cultured cases [12]. Locally, the confirmation of *S. milleri* was predictive of post-operative abscess formation (29% of cases with milleri *Streptococcus spp.* cultured) further highlighting the added benefit of culture results, similar to previous studies [11, 12].

The clinical significance of intra-operative *Pseudomonas *spp*.* is not fully understood. Unlike in adult appendicitis, paediatric cases persistently report on the presence of *Pseudomonas *spp*.* with prevalence ranging from 0 to 29% of all cultures, similar to the rate found in this local study (21%) [19, 26–28]. Identification of *Pseudomonas *spp*.* cultures resulted in an extended LOS but had no impact on confirmed rates of abscess formation or re-admission. Many patients were managed with gentamicin in combination with co-amoxiclav, with target beta-lactam therapy (e.g. piperacillin/tazobactam) reserved for children with uncontrolled systemic infection or confirmed abscess formation [19]. Theodorou et al. describe a similar rate of surgical site infection in both *Pseudomonas *spp*.* positive and negative patients [19], while other studies suggest that surgical site infection is higher with *Pseudomonas *spp*.* where prophylactic coverage of this pathogen was often lacking [25]. In our local practice, empiric antipseudomonal antimicrobial surgical prophylaxis is recommended (gentamicin) and as such may mitigate against some of the possible *Pseudomonas *spp*.* complications. A control of non-pseudomonal surgical prophylaxis would be required to accurately determine the true pathogenicity of this pathogen in complex paediatric appendicitis.

### Limitations

This study had a retrospective design, and microbiological data was not routinely collected for all patients. In some cases of perforated appendicitis, intra-operative cultures were not available, and this selective nature may over-estimate bacterial involvement by association with more severe presentations. The study is limited to two hospital sites and may not reflect epidemiology of other regional areas. Local surgical practice, empirical antibacterial prescribing and antimicrobial resistance patterns may vary with other external centres [24].

Intra-operative cultures were not available for all children due to the retrospective nature of the study design. Cultures were more likely to be taken in children with evidence of local peritonitis or systemic signs of sepsis. Thus, the studied cohort is not reflective of all children requiring an appendectomy, and our results are confounded by more severe surgical cases.

Intra-operative culturing is commonly utilised; however, correlating positive cultures with confirmed invasive infections is challenging. Inference of outcomes from initial culturing may, therefore, be inaccurate. Post-operative sampling is uncommon in this practice, as successful culturing using traditional microbiological methods post exposure of systemic antibacterials is problematic. Serial invasive sampling using molecular microbiological may provide more accurate epidemiological data, but unfortunately, is not widely available in our practice.

## Conclusion

This study confirmed the utility of intra-operative cultures for complex paediatric appendectomies. Due to increasing *Enterobacterales* resistance, the possibility of abscess formation post-operatively with some *Streptococcus *spp*.* and the presence of *Pseudomonas *spp., culture results may help guide clinicians’ judgement when deciding to alter post-operative treatment to achieve clinical stability. Whilst beta-lactam combinations are widely recommended for surgical prophylaxis, locally we continue to recommend the addition of an aminoglycoside to extend activity against resistant *Enterobacterales* and *Pseudomonas *spp*.* Further prospective studies are required to further understand the pathogenicity of commonly isolated pathogens to support tailored treatment plans.

## Data Availability

The datasets analysed during the current study and further details on gaining access to the intervention reported within this study are available from the senior author (SH stephen.hughes10@nhs.net) on reasonable request, as long as this meets local ethics and research governance criteria.

## References

[1] Gadiparthi R, Waseem M (2022). Pediatric appendicitis.

[2] Rentea RM, Peter SDS, Snyder CL (2017). Pediatric appendicitis: state of the art review. Pediatr Surg Int.

[3] Alloo J, Gerstle T, Shilyansky J, Ein SH (2004). Appendicitis in children less than 3 years of age: a 28-year review. Pediatr Surg Int.

[4] Lee SL, Stark R, Yaghoubian A, Shekherdimian S, Kaji A (2011). Does age affect the outcomes and management of pediatric appendicitis?. J Pediatr Surg.

[5] Pearl RH, Hale DA, Molloy M, Schutt DC, Jaques DP (1995). Pediatric appendectomy. J Pediatr Surg.

[6] Jones MW, Lopez RA, Deppen JG, Appendicitis. *StatPearls* Treasure Island (FL): StatPearls Publishing; 2022.

[7] Brennan GDG (2006). Pediatric appendicitis: pathophysiology and appropriate use of diagnostic imaging. CJEM.

[8] Roberts JP (1988). Quantitative bacterial flora of acute appendicitis. Arch Dis Child.

[9] Rautio M, Saxén H, Siitonen A, Nikku R, Jousimies-Somer H (2000). Bacteriology of histopathologically defined appendicitis in children. Pediatr Infect Dis J.

[10] Madden NP, Hart CA (1985). Streptococcus milleri in appendicitis in children. J Pediatr Surg.

[11] Leeuwenburgh MMN, Monpellier V, Vlaminckx BJM, Go, Peter MNYH (2012). Streptococcus milleri in intraabdominal abscesses in children after appendectomy: incidence and course. J Pediatr Surg.

[12] Subramanian T, Jerome E, Jones I, Jester I (2018). Streptococcus anginosus is associated with postoperative intraabdominal collections in appendicitis. J Pediatr Surg.

[13] Andrey V, Crisinel P, Prod’hom G, Croxatto A, Joseph J (2019). Impact of co-amoxicillin-resistant Escherichia coli and pseudomonas aeruginosa on the rate of infectious complications in paediatric complicated appendicitis. Swiss Med Wkly.

[14] Reinisch A, Malkomes P, Habbe N, Bechstein WO, Liese J (2017). Bad bacteria in acute appendicitis: rare but relevant. Int J Colorectal Dis.

[15] Potts S, Hamilton J, Murchison L, Carachi R (2014). G72 (P) management of acute appendicitis in children. Arch Dis Child.

[16] Rafiq MS, Khan MM, Khan A, Jan H (2015). Evaluation of postoperative antibiotics after non-perforated appendectomy. J Pak Med Assoc.

[17] Taleb M, Nardi N, Arnaud A, Costet N, Donnio P, Engrand C (2018). Simplification of first-line antibacterial regimen for complicated appendicitis in children is associated with better adherence to guidelines and reduced use of antibiotics. Int J Antimicrob Agents.

[18] Fallon SC, Hassan SF, Larimer EL, Rodriguez JR, Brandt ML, Wesson DE (2013). Modification of an evidence-based protocol for advanced appendicitis in children. J Surg Res.

[19] Theodorou CM, Stokes SC, Hegazi MS, Brown EG, Saadai P (2021). Is pseudomonas infection associated with worse outcomes in pediatric perforated appendicitis?. J Pediatr Surg.

[20] GOV.UK. Standards for microbiology investigations (UK SMI) https://www.gov.uk/government/collections/standards-for-microbiology-investigations-smi [Accessed May 11, 2022].

[21] EUCAST. EUCAST: MIC and zone distributions and ECOFFs. https://www.eucast.org/mic_distributions_and_ecoffs/.

[22] Foo FJ, Beckingham IJ, Ahmed I (2008). Intra-operative culture swabs in acute appendicitis: a waste of resources. Surgeon: J Royal Colleges Surg Edinb Irel.

[23] Son JT, Lee GC, Kim HO, Kim T, Lee D, Lee SR (2020). Routine intraoperative bacterial culture may be needed in complicated appendicitis. Annals of Coloproctology.

[24] Hu A, Li J, Vacek J, Bouchard M, Ingram M, McMahon M (2021). Antibiotic resistance is common in the cultures of intraabdominal abscess drainage after appendectomy. J Pediatr Surg.

[25] Chen C, Chen Y, Pu H, Tsai C, Chen W, Lin C (2012). Bacteriology of acute appendicitis and its implication for the use of prophylactic antibiotics. Surg Infect.

[26] Obinwa O, Casidy M, Flynn J (2014). The microbiology of bacterial peritonitis due to appendicitis in children. Ir J Med Sci.

[27] Boueil A, Guégan H, Colot J, D’Ortenzio E, Guerrier G (2015). Peritoneal fluid culture and antibiotic treatment in patients with perforated appendicitis in a Pacific Island. Asian J Surg.

[28] Turel O, Mirapoglu SL, Yuksel M, Ceylan A, Gultepe BS (2019). Perforated appendicitis in children: antimicrobial susceptibility and antimicrobial stewardship. J Global Antimicrob Resist.

